# Delayed discharges revisited: impact of a liaison post on patients' transition from ICU to ward care

**DOI:** 10.1186/cc11120

**Published:** 2012-03-20

**Authors:** J Mellinghoff, P O'Shea, D Dawson, J Ball, A Rhodes, M Grounds

**Affiliations:** 1 St George's Healthcare NHS Trust, London, UK

## Introduction

This audit reviewed the discharge process of patients from an adult general ICU to the general wards before and after the introduction of a liaison nurse post over a 3-year, 3-month time period.

## Methods

The audit utilised routinely collected retrospective data from a 17-bed ICU. We examined the impact of a liaison post on the length of delays on discharge of patients from the ICU to the general wards.

## Results

**Figure 1:**
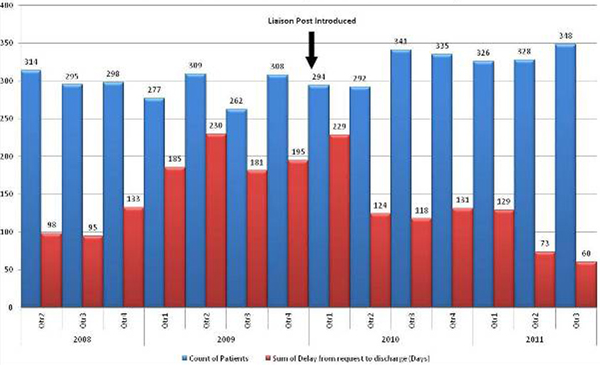
**Number of ward discharges and accumulated delay**.

The study period was from April 2008 until June 2011 with the start date of the liaison nurse post in January 2010. Overall, there were 4,327 patient discharges to hospital wards (before group = 2,063, after group = 2,264). The odds of experiencing a delay in discharge >4 hours were 3.2-fold higher in the before group compared to the after group (95% CI = 2.808 to 3.717, *P *< 0.0001). Accumulated discharge delays decreased by 23% from 1,116 (before group) to 864 days (after group) despite an increase in patient turnover of 10% (*n = *201). The median delay time was 7.2 hours (IQR 5.0 hours, 10.4 hours) in the before group and 5.3 hours in the after group (IQR 2.7 hours, 9.0 hours). See Figure 1.

## Conclusion

Our analysis suggests that the introduction of a liaison nurse post within intensive care significantly reduced the length of delays in the discharge process despite an increase in patient turnover.

